# Circulating Interleukin-6 Mediates PM_2.5_-Induced Ovarian Injury by Suppressing the PPARγ Pathway

**DOI:** 10.34133/research.0538

**Published:** 2024-12-05

**Authors:** Yingying Chen, Jinjin Zhang, Tianyu Zhang, Yaling Wu, Yueyue Xi, Tong Wu, Mo Li, Yan Li, Su Zhou, Mingfu Wu, Shixuan Wang

**Affiliations:** ^1^ Department of Obstetrics and Gynecology, National Clinical Research Center for Obstetrics and Gynecology, Tongji Hospital, Tongji Medical College, Huazhong University of Science and Technology, Wuhan, China.; ^2^ Department of Gynecology, The First Affiliated Hospital of Zhengzhou University, Zhengzhou University, Zhengzhou, China.; ^3^ Key Laboratory of Cancer Invasion and Metastasis (Ministry of Education), Hubei Key Laboratory of Tumor Invasion and Metastasis, Tongji Hospital, Tongji Medical College, Huazhong University of Science and Technology, Wuhan, China.

## Abstract

Exposure to airborne fine particulate matter (PM_2.5_) is strongly associated with poor fertility and ovarian damage. However, the mechanism underlying this remains largely unclear. Here, we found that PM_2.5_ markedly impaired murine ovarian reserve, decreased hormone levels, and aggravated ovarian inflammation. Circulating interleukin-6 (IL-6) was elevated in PM_2.5_-exposed mice and was further confirmed to mediate this damage by IL-6 recombinant protein intervention. PM_2.5_ exposure led to increased alveolar macrophage infiltration in the lungs. However, alveolar macrophage clearance with clodronate liposomes could not fully reverse the elevated IL-6 levels and ovarian injury, suggesting that alveolar macrophages were probably not the only source of circulating IL-6. Further experiments indicated that IL-6 mainly targeted ovarian theca–interstitial cells and impaired testosterone synthesis via suppressing the peroxisome proliferator-activated receptor γ (PPARγ) pathway. In addition, apoptosis of granulosa cells and restriction of follicular growth were observed in co-cultures with IL-6-treated theca–interstitial cells, which could be further reversed by the PPARγ agonist. Moreover, IL-6-neutralizing antibodies ameliorated PM_2.5_-induced ovarian damage. Notably, increased levels of circulating IL-6 were observed in premature ovarian aging patients and were inversely associated with their ovarian function. In summary, our findings offer a mechanistic explanation for PM_2.5_-induced ovarian dysfunction and verify IL-6 as a biomarker and potential therapeutic target.

## Introduction

Fine particulate matter (PM_2.5_) pollution is an unavoidable environmental problem worldwide, both presently and in the future. Due to its wide distribution, small particle size, and complex composition, its harmful health effects are of increasing concern [1,2]. The International Agency for Research on Cancer classified PM_2.5_ as a human class I carcinogen in 2013 [3], and it was responsible for up to 1.8 million deaths in 13,160 urban areas worldwide in 2019 [4]. PM_2.5_ has been linked to various adverse health outcomes, including respiratory and cardiovascular diseases [5–7], stroke [6–8], Alzheimer’s disease [9], diabetes [6,10], and obesity [11,12]. Consequently, the multi-organ injury caused by PM_2.5_ has attracted considerable attention.

Accumulating evidence suggests that PM_2.5_ exposure is associated with poor fertility [13,14] and adverse pregnancy outcomes [15,16]. A large prospective cohort study showed that nurses exposed to PM_2.5_ in midlife experienced menopause at an earlier age [17]. The antral follicle count (AFC) is widely used clinically to assess ovarian reserve. Data from 632 women at the Massachusetts Reproductive Center showed a negative association between PM_2.5_ concentration and AFC [18]. Animal studies have suggested that exposure to PM_2.5_ affects ovarian hormone secretion and oocyte maturation, and metal imbalance and steroid synthesis disruption are involved in the process [19]. However, the detailed mechanisms and key molecules responsible for systematic PM_2.5_-exposure-induced ovarian injury remain largely unclear, hampering the development of strategies to protect ovarian function.

Our prior research has shown that the soluble fractions of PM_2.5_ exacerbated ovarian damage, which is associated with increased oxidative stress and inflammation [20]. However, direct evidence of PM_2.5_ deposition in ovarian tissues is lacking. The observation and quantification of real-time PM_2.5_ deposition in the ovaries are hindered by the limited blood flow to these tissues. Previous studies have indicated that PM_2.5_ exposure can cause systemic inflammation [21–23], a condition closely linked to ovarian dysfunction [24,25]. Elevated concentrations of circulating cytokines, including interleukin-6 (IL-6), interleukin-1 beta, interleukin-10, interleukin-12, interleukin-17, and tumor necrosis factor α, are well-recognized indicators of systemic inflammation. Consequently, we hypothesize that these elevated levels of circulating inflammatory cytokines may contribute to PM_2.5_-induced ovarian damage.

This study verified the ovarian damage caused by PM_2.5_, identified the major mediators, and elucidated the target cells and underlying molecular mechanisms. Notably, we demonstrated the therapeutic potential of IL-6-neutralizing antibodies (IL-6 Abs) against such damage. Moreover, we observed a negative correlation between IL-6 levels and ovarian function in women. Our findings provide novel insights into how PM_2.5_ damages the ovaries and confirm IL-6 as a key mediator and potential therapeutic target for PM_2.5_-induced ovarian damage.

## Results

### Damages of the ovarian reserve and steroid synthesis induced by PM_2.5_ exposure

**Fig. 1.**
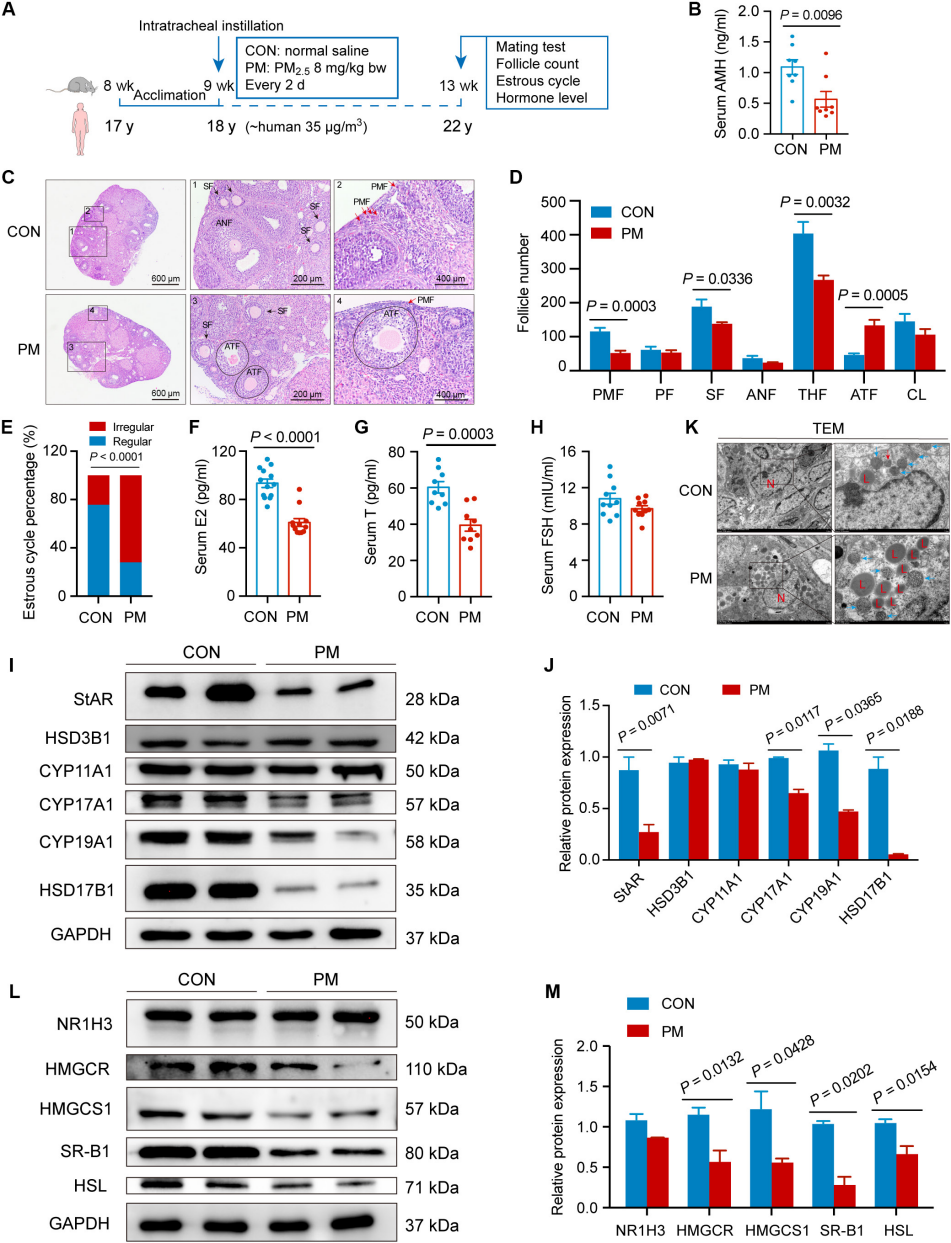
PM2.5 exposure induced ovarian injury. (A) Scheme of the animal study for assessing the effect of PM2.5 exposure on ovarian function. (B) The serum anti-Müllerian hormone (AMH) level in PM2.5-exposed mice (n = 8 mice/group, 2-tailed Student t test). (C) Ovarian hematoxylin and eosin (H&E) staining image from PM2.5-exposed mice. (D) Follicle counting results according to ovary serial sections (n = 5 mice/group, 2-tailed Student t test). (E) The proportion of regular or irregular estrous cycles in PM2.5-exposed mice (n = 8 mice/group, chi-square test). (F to H) The serum hormone level of mice exposed to PM2.5 (n = 9 to 13 mice/group, 2-tailed Student t test). (I and J) Protein expression of the hormone synthesis rate-limiting enzyme in ovaries from PM2.5-exposed mice detected by Western blot (n = 3 biological replicates/group, 2-tailed Student t test). (K) Transmission electron microscopy of ovarian tissue from mice exposed to PM2.5. Blue arrows indicate mitochondria. (L and M) Protein expression of cholesterol-metabolism-related genes in ovaries from PM2.5-exposed mice detected by Western blot (n = 3 biological replicates/group, 2-tailed Student t test). PMF, primordial follicle; PF, primary follicle; SF, secondary follicle; ANF, antral follicle; THF, total healthy follicle; ATF, atretic follicle; CL, corpus luteum; E2, estradiol; T, testosterone; FSH, follicle-stimulating hormone; N, nucleus; L, lipid droplet.

To evaluate the effects of exposure to PM_2.5_ on ovarian function, C57BL/6 mice were exposed to PM_2.5_ suspension (8 mg/kg body weight [bw]) via intratracheal dripping every 2 d (equivalent to the human exposure concentration of 35 μg/m^3^) (Fig. 1A). The serum anti-Müllerian hormone (AMH) concentration significantly decreased after exposure to PM_2.5_ (Fig. 1B). Compared with those of the control (CON) group, the number of primordial follicles, secondary follicles, and total healthy follicles in the PM_2.5_-treated group were significantly decreased (Fig. 1C and D), while the number of atretic follicles was significantly increased (Fig. 1D). The percentages of irregular estrous cycles in the CON and PM groups were 25% and 72%, respectively (Fig. 1E). As shown in Fig. 1F to H, exposure to PM_2.5_ significantly decreased the concentration of serum estradiol (E2) and testosterone (T), whereas no significant differences in serum follicle-stimulating hormone (FSH) levels were observed between the CON and PM groups. These results indicate that PM_2.5_ exposure diminishes the ovarian reserve and disrupts steroid synthesis.

We examined the expression of related genes to elucidate the molecular mechanisms underlying the disrupted steroid synthesis. Western blot analysis showed that exposure to PM_2.5_ significantly decreased the expression of StAR, CYP17A1, CYP19A1, and HSD17B1 (Fig. 1I and J). Free cholesterol is directly used for steroid hormone synthesis, whereas cholesteryl esters, stored in lipid droplets, cannot be directly used for hormone synthesis [26]. Transmission electron microscopy (TEM) revealed that PM_2.5_ induced a large accumulation of lipid droplets in theca–interstitial cells (TICs) (Fig. 1K), potentially affecting hormone synthesis. Our results further demonstrated that exposure to PM_2.5_ down-regulated the expression of cholesterol-metabolism-related genes, including HMGCR, HMGCS1, SR-B1, and HSL. Together, these findings suggest that PM_2.5_ exposure disrupts steroid biosynthesis in murine ovaries.

### Elevation of circulating IL-6 mediated PM_2.5_-induced ovarian injury

**Fig. 2.**
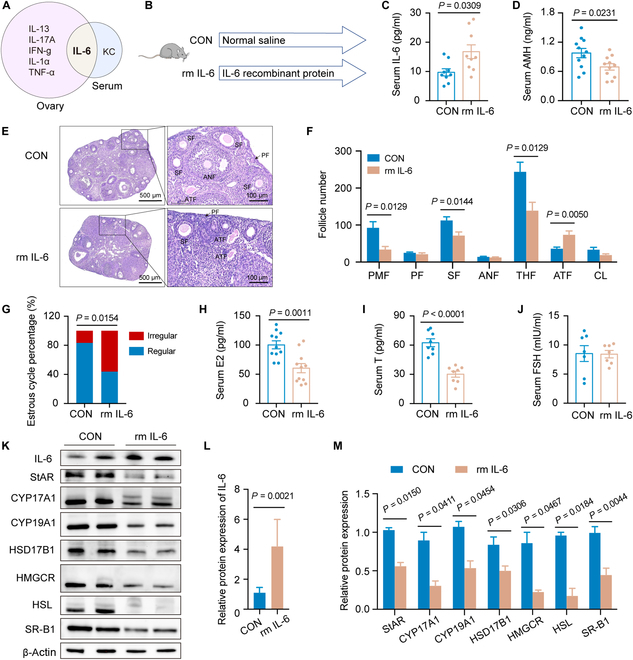
Circulating interleukin-6 (IL-6) mediated PM_2.5_-induced ovarian injury. (A) Venn plots of elevated cytokines in the circulation and ovaries of mice exposed to PM_2.5_. (B) Scheme of the animal study for assessing the effect of IL-6 on ovarian function. (C) The serum IL-6 levels of mice upon administration of IL-6 recombinant protein (rm IL-6) (*n* = 9 mice/group, 2-tailed Student *t* test). (D) The serum AMH level of mice treated with rm IL-6 (*n* = 11 mice/group, 2-tailed Student *t* test). (E) Ovarian H&E staining images of mice treated with rm IL-6. (F) Follicle counting results according to ovary serial sections (*n* = 5 mice/group, 2-tailed Student *t* test). (G) The proportion of regular or irregular estrous cycles of mice treated with rm IL-6 (*n* = 11 mice/group, chi-square test). (H to J) The serum hormone level of mice treated with rm IL-6 (*n* = 7 to 11 mice/group, 2-tailed Student *t* test). (K to M) Protein expression of hormone-synthesis-related genes in ovaries from rm IL-6-treated mice detected by Western blot (*n* = 3 biological replicates/group, 2-tailed Student *t* test). rm IL-6, IL-6 recombinant protein; KC, keratinocyte-derived chemokine.

For the mechanism investigation of PM_2.5_-induced ovarian injury, RNA sequencing analysis was conducted. Gene Ontology enrichment analysis showed that the genes down-regulated were enriched in cellular hormone metabolic processes mainly (Fig. S1A), whereas the up-regulated genes were primarily concentrated in the acute phase response and inflammatory response (Fig. S1B). These findings suggested that inflammation may be involved in PM_2.5_-induced ovarian injury. Further examination of inflammatory factors in the ovaries utilizing the Luminex liquid suspension chip showed an increase in interleukin-13, IL-6, interleukin-17A, and other factors in PM_2.5_-exposed ovaries (Fig. S1C and D). As PM_2.5_ is known to cause systemic inflammation [21–23], the elevated levels of circulating cytokines induced by PM_2.5_ may be an important factor contributing to ovarian inflammation. Serum Luminex liquid suspension chip analysis further revealed a significant increase in circulating IL-6 and keratinocyte-derived chemokine after exposure to PM_2.5_ (Fig. S1E and F). Combining the analyses of ovarian and serum cytokines, it was evident that IL-6 levels were significantly elevated (Fig. 2A), which suggests its key role in mediating PM_2.5_-induced ovarian injury.

To determine whether circulating IL-6 mediates PM_2.5_-induced ovarian injury, mice were treated with IL-6 recombinant protein (rm IL-6) (Fig. 2B). The results showed that rm IL-6 increased serum IL-6 levels (Fig. 2C); decreased serum AMH levels (Fig. 2D); reduced the number of primordial follicles, secondary follicles, and total healthy follicles; and increased the number of atretic follicles (Fig. 2E and F). The proportion of irregular estrous cycles was significantly increased. The levels of serum E2 and T were lower in the rm IL-6 group than in the CON group (Fig. 2H and I). In contrast, serum FSH levels were not significantly different between the 2 groups. The expression of hormone-synthesis-related genes also decreased (Fig. 2K to M). In summary, these findings imply that circulating IL-6 may mediate PM_2.5_-induced ovarian injury.

### Alveolar macrophages were probably not the major source of circulating IL-6

Previous research studies have revealed that acute PM_2.5_ exposure induces IL-6 secretion due to alveolar macrophages, resulting in elevated circulating IL-6 [27–30]. To clarify whether circulating IL-6 was derived from alveolar macrophages, we identified the expression of IL-6 in alveolar macrophages and conducted alveolar macrophage clearance experiments. Hematoxylin and eosin (H&E) staining of murine lungs showed that PM_2.5_ exposure led to a disrupted alveolar structure, a thickened alveolar septa, and increased immune cell infiltration (Fig. S2A). Immunohistochemistry (IHC) staining further suggested that PM_2.5_ induced alveolar macrophage infiltration (Fig. S2B to E). These results suggest that alveolar macrophages are a potential source of elevated circulating IL-6 levels.

**Fig. 3.**
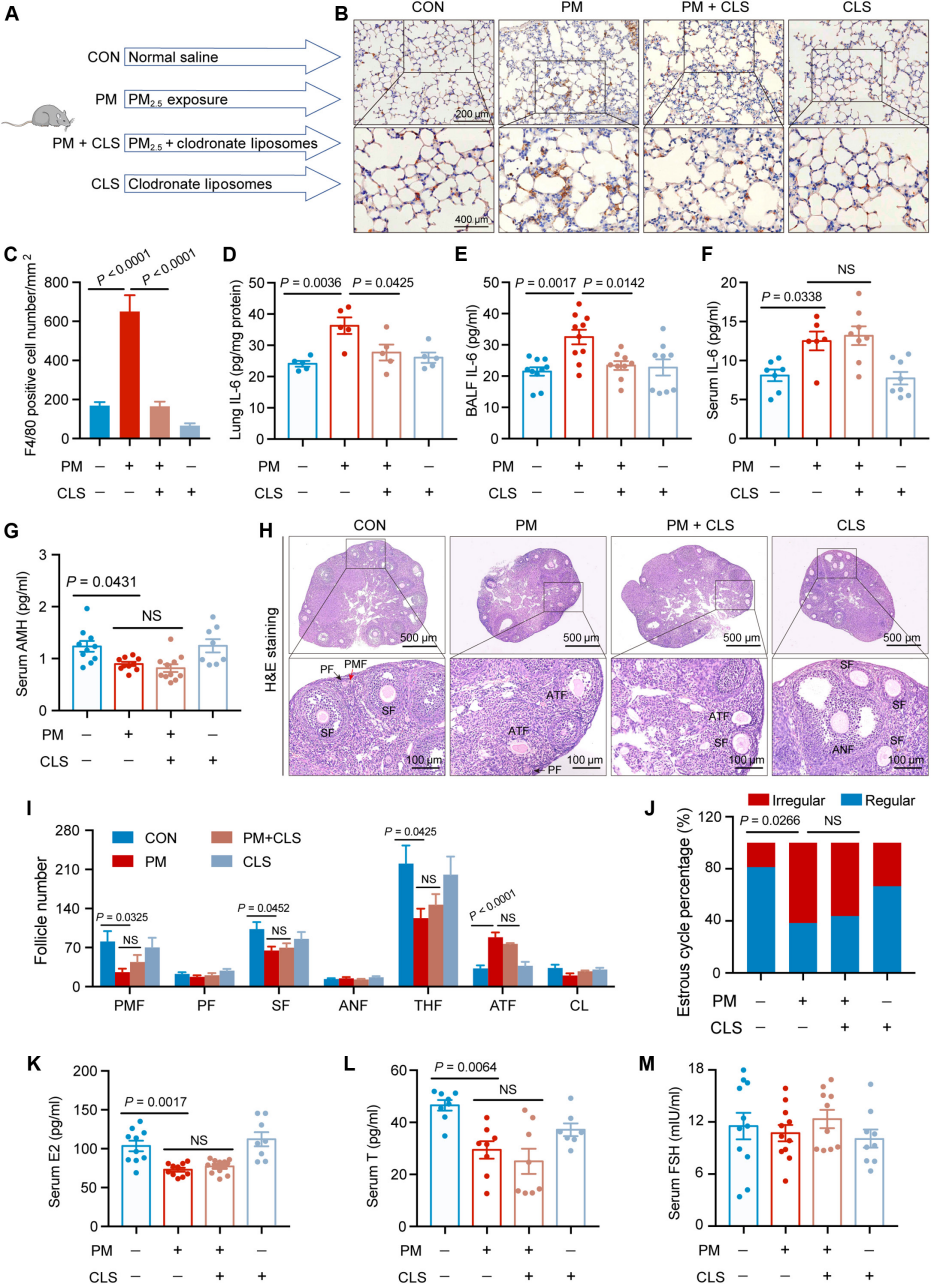
Alveolar macrophages were probably not the major source of circulating IL-6. (A) Scheme of the animal study for assessing whether alveolar macrophage clearance can reverse PM_2.5_-induced elevated circulating IL-6 and ovarian injury. (B) Representative images of anti-F4/80 staining in lungs from mice exposed to PM_2.5_, with or without clodronate liposome (CLS) administration. (C) The number of macrophages in murine lungs according to anti-F4/80 staining (*n* = 3 mice/group, one-way analysis of variance [ANOVA]). (D to F) The IL-6 levels in the lung (*n* = 5 mice/group), bronchoalveolar lavage fluid (BALF) (*n* = 9 to 10 mice/group), and serum (*n* = 6 to 8 mice/group) of mice exposed to PM_2.5_ with or without CLS administration (one-way ANOVA). (G) The serum AMH level of mice exposed to PM_2.5_ with or without CLS administration (*n* = 8 to 11 mice/group, one-way ANOVA). (H) Ovarian H&E staining images of mice exposed to PM_2.5_ with or without CLS administration. (I) Follicle counting results according to ovary serial sections (*n* = 5 mice/group, one-way ANOVA). (J) The proportion of regular or irregular estrous cycles of mice exposed to PM_2.5_ with or without CLS administration (*n* = 11 mice/group, chi-square test). (K to M) The serum hormone level of mice exposed to PM_2.5_ with or without CLS administration (*n* = 7 to 11 mice/group, one-way ANOVA). CLS, clodronate liposomes (one-way ANOVA).

To verify this hypothesis, we performed alveolar macrophage clearance with clodronate liposomes (CLS) and investigated whether it could reverse the elevated circulating IL-6 levels and ovarian injury caused by PM_2.5_ (Fig. 3A). The results demonstrated that CLS reversed PM_2.5_-induced alveolar macrophage infiltration and elevated IL-6 levels in lung tissues (Fig. 3B to E); however, a little change was observed in circulating IL-6 levels (Fig. 3F). Furthermore, PM_2.5_-induced ovarian injury was not alleviated by CLS treatment (Fig. 3G to M). Therefore, these results indicate that alveolar macrophages are probably not a major source of circulating IL-6.

### Disruption of T synthesis in TICs by IL-6 through the PPARγ pathway

**Fig. 4.**
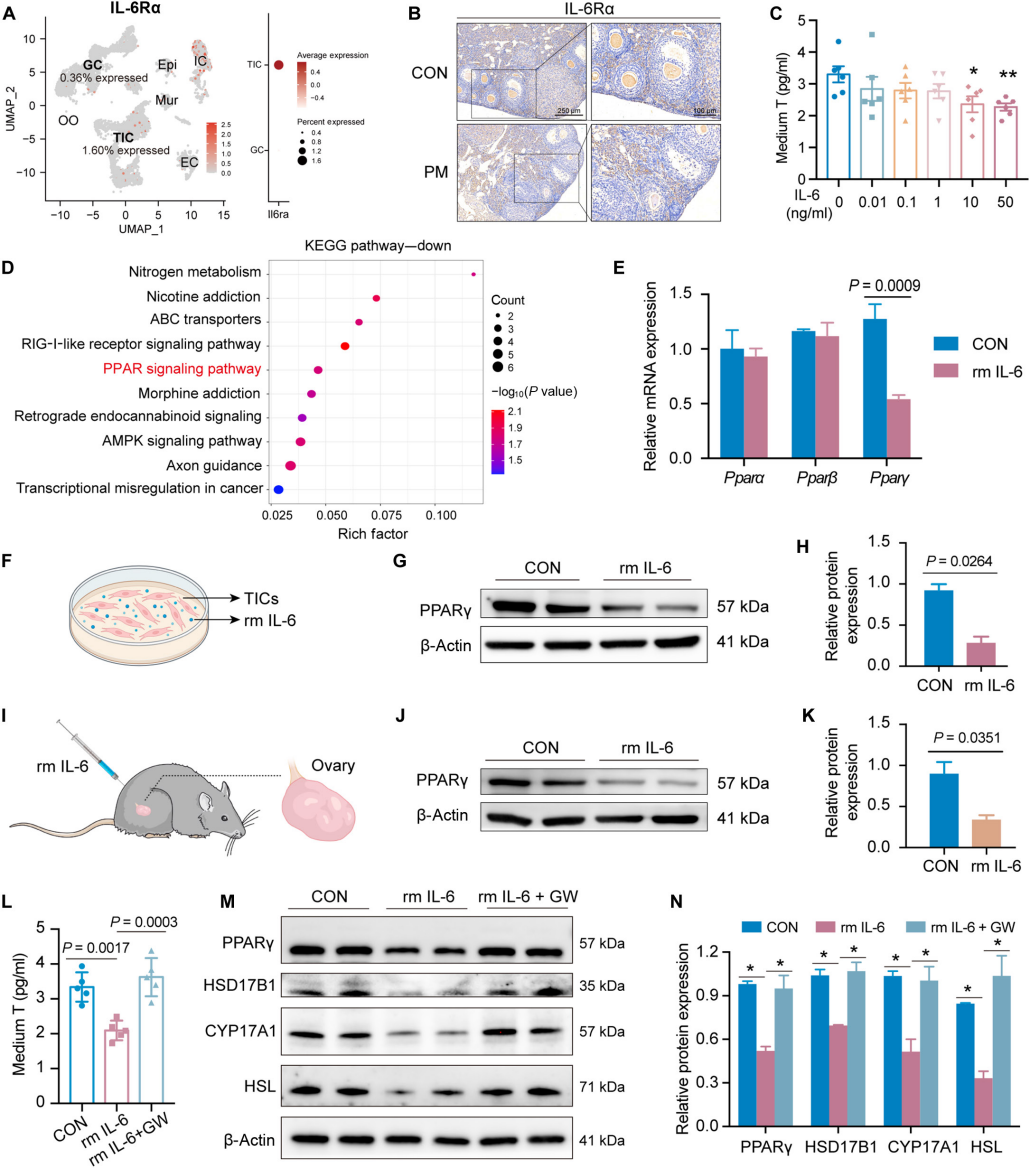
IL-6 affected testosterone synthesis in theca–interstitial cells (TICs) through the peroxisome proliferator-activated receptor γ (PPARγ) pathway. (A) Uniform manifold approximation and projection (UMAP) plot and dot plot of IL-6Rα expression in identified cell populations in the murine single-cell dataset. (B) Representative images of anti-IL-6Rα staining in murine ovaries. (C) The testosterone level in the medium of TICs treated with graded concentrations of rm IL-6 (*n* = 6 biological replicates/group, one-way ANOVA). (D) Top 10 pathways of down-regulated genes by Kyoto Encyclopedia of Genes and Genomes (KEGG) in TICs treated with 50 ng/ml rm IL-6. (E) Relative messenger RNA (mRNA) expression of genes related to the peroxisome proliferator-activated receptor (PPAR) pathway in TICs treated with rm IL-6 (*n* = 3 biological replicates/group, 2-tailed Student *t* test). (F to H) Protein expression of PPARγ in TICs treated with rm IL-6 detected by Western blot (*n* = 3 biological replicates/group, 2-tailed Student *t* test). (I to K) Protein expression of PPARγ in ovaries from mice treated with rm IL-6 detected by Western blot (*n* = 3 biological replicates/group, 2-tailed Student *t* test). (L) The testosterone level in the medium of TICs treated with rm IL-6 combined with or without the PPARγ agonist GW1929 (*n* = 5 biological replicates/group, one-way ANOVA). (M and N) Protein expression of hormone-synthesis-related genes in TICs treated with rm IL-6 combined with or without GW1929 detected by Western blot (*n* = 3 biological replicates/group, one-way ANOVA). ^*^P < 0.05. GW, PPARγ agonist GW1929.

The molecular mechanism of IL-6-mediated ovarian injury caused by PM_2.5_ was further investigated. Single-cell RNA sequencing of murine ovaries showed that the IL-6 receptor (IL-6Rα) was highly expressed in TICs and almost absent in granulosa cells (GCs) (Fig. 4A). The IHC of ovarian tissues further indicated that IL-6Rα was specifically expressed in TICs (Fig. 4B). Therefore, we hypothesized that TICs are direct targets of IL-6.

We then explored the role of IL-6 on TICs. Mouse primary TICs treated with rm IL-6 showed no obvious effect on cell proliferation (Fig. S3A and B); however, they reduced T synthesis and decreased hormone-synthesis-related gene expression—*Hsd17b1*, *Cyp17a1*, and *Hsl* (Fig. 4C and Fig. S3C to L). We further explored the molecular mechanisms by which IL-6 affects T synthesis in TICs. Transcriptome sequencing revealed that the genes down-regulated in IL-6-treated TICs were enriched in the RIG-I-like signaling pathway, the peroxisome proliferator-activated receptor (PPAR) signaling pathway, the adenosine 5′-monophosphate-activated protein kinase signaling pathway, etc. (Fig. 4D). Among them, the PPAR pathway is involved in the regulation of lipid metabolism, adipocyte differentiation, glycogen heterodimerization, and cell survival [31,32], which aroused our interest for further study. Specifically, IL-6 was found to result in a notable reduction in peroxisome proliferator-activated receptor γ (PPARγ) transcript and protein expression in TICs and ovaries (Fig. 4E to K). Additionally, a PPARγ agonist, GW1929, reversed the IL-6-induced reduction of T and expression of hormone-synthesis-related genes HSD17B1, CYP17A1, and HSL (Fig. 4L to N). These findings suggest that IL-6 disrupts T synthesis in TICs through the PPARγ pathway.

### Inhibition of GC E2 synthesis and follicular growth by IL-6-treated TICs

**Fig. 5.**
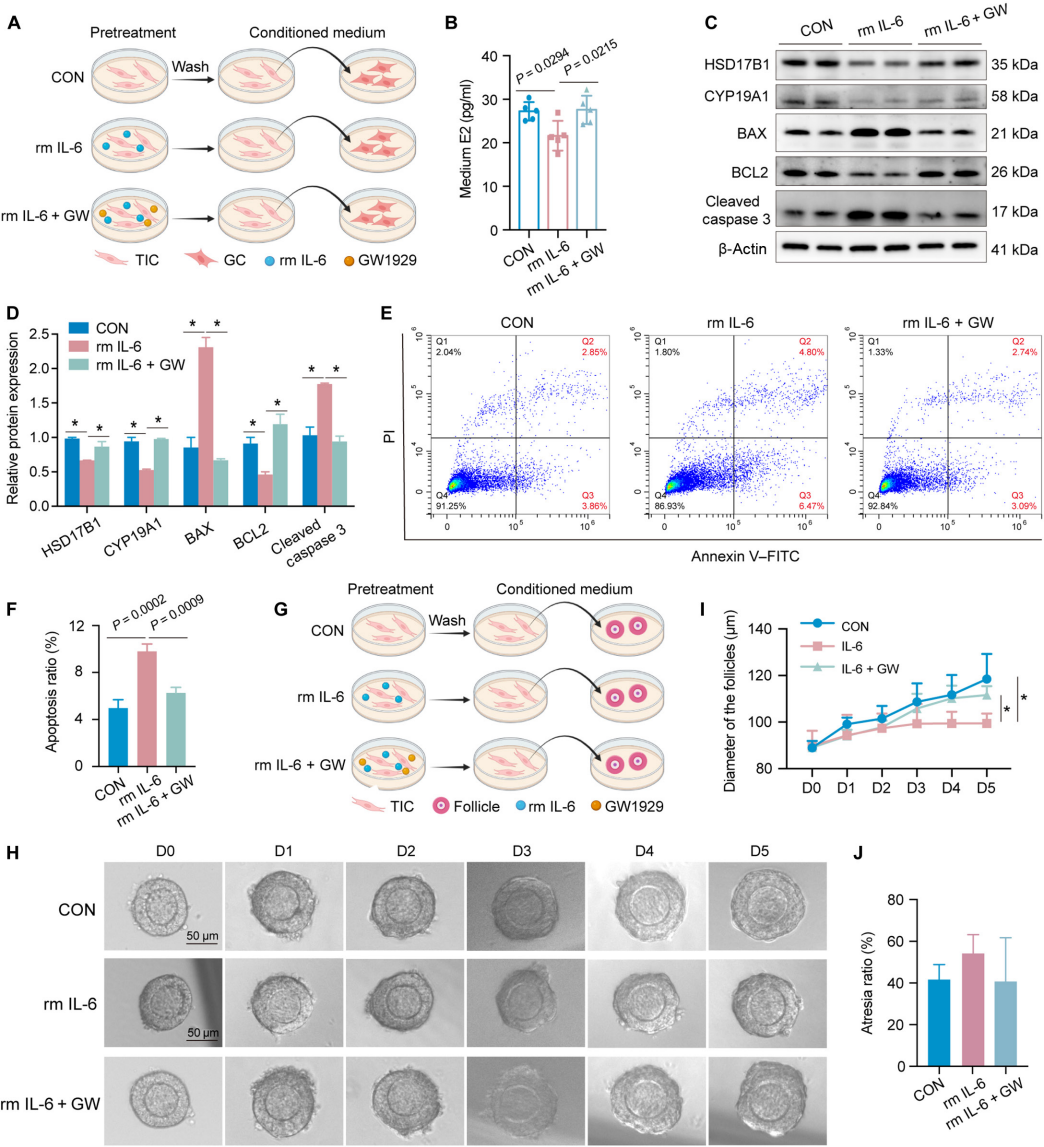
IL-6 decreased granulosa cell (GC) estradiol synthesis and restricted follicular growth through TICs. (A) Scheme of the experiment on GC intervention. In brief, TICs were pretreated with 50 ng/ml rm IL-6 with or without GW1929 for 24 h. Afterward, they were washed thrice with phosphate-buffered saline (PBS) and incubated in a complete medium without IL-6 or GW1929 for another 24 h. Culture supernatants from the TICs were collected and used as a conditioned medium for GC culture. (B) The estradiol level in the culture supernatants of GCs treated with conditioned medium (*n* = 5 biological replicates/group, one-way ANOVA). (C and D) Protein expression of hormone-synthesis-related genes and apoptosis-related genes in GCs treated with a conditioned medium detected by Western blot (*n* = 3 biological replicates/group, one-way ANOVA). (E and F) Apoptosis of GCs treated with a conditioned medium detected by flow cytometry (*n* = 3 biological replicates/group, one-way ANOVA). (G) Scheme of the experiment on follicular intervention. In brief, TICs were pretreated with 50 ng/ml rm IL-6 with or without GW1929 for 24 h. Afterward, they were washed thrice with PBS and incubated in a complete medium without IL-6 or GW1929 for another 24 h. Culture supernatants from the TICs were collected and used as a conditioned medium for follicle culture. (H) Representative images of follicles treated with a conditioned medium. (I) The diameter of follicles treated with a conditioned medium (*n* = 3 biological replicates/group, 2-way ANOVA). (J) The atresia ratio of follicles treated with a conditioned medium (*n* = 3 biological replicates/group, one-way ANOVA). ^*^P < 0.05.

According to the 2-cell theory of hormone synthesis, androgens synthesized by TICs are the raw materials for estrogen biosynthesis by GCs [33–35]. To clarify the effect of IL-6 on hormone synthesis by GCs, GCs were co-cultured with a conditioned medium of IL-6-treated TICs (Fig. 5A). We found that the conditioned medium resulted in reduced E2 synthesis and decreased HSD17B1 and CYP19A1 expression in GCs (Fig. 5B to D). It also induced GC apoptosis (Fig. 5C to F), which partially explains the cause of follicular atresia caused by PM_2.5_.

In order to ascertain the impact of IL-6 on follicular growth, we co-cultured preantral follicles with a conditioned medium of IL-6-treated TICs (Fig. 5G). The results showed that IL-6-treated TICs restricted follicular growth (Fig. 5H and I); however, no significant effect was observed on follicular atresia (Fig. 5J), which might be related to the duration of the intervention. Furthermore, the PPARγ agonist reversed the effects induced by IL-6 (Fig. 5A to I). These results suggest that IL-6 decreases GC E2 synthesis and restricts follicular growth via TICs.

### Alleviation of PM_2.5_-induced ovarian injury by Il-6 Abs

**Fig. 6.**
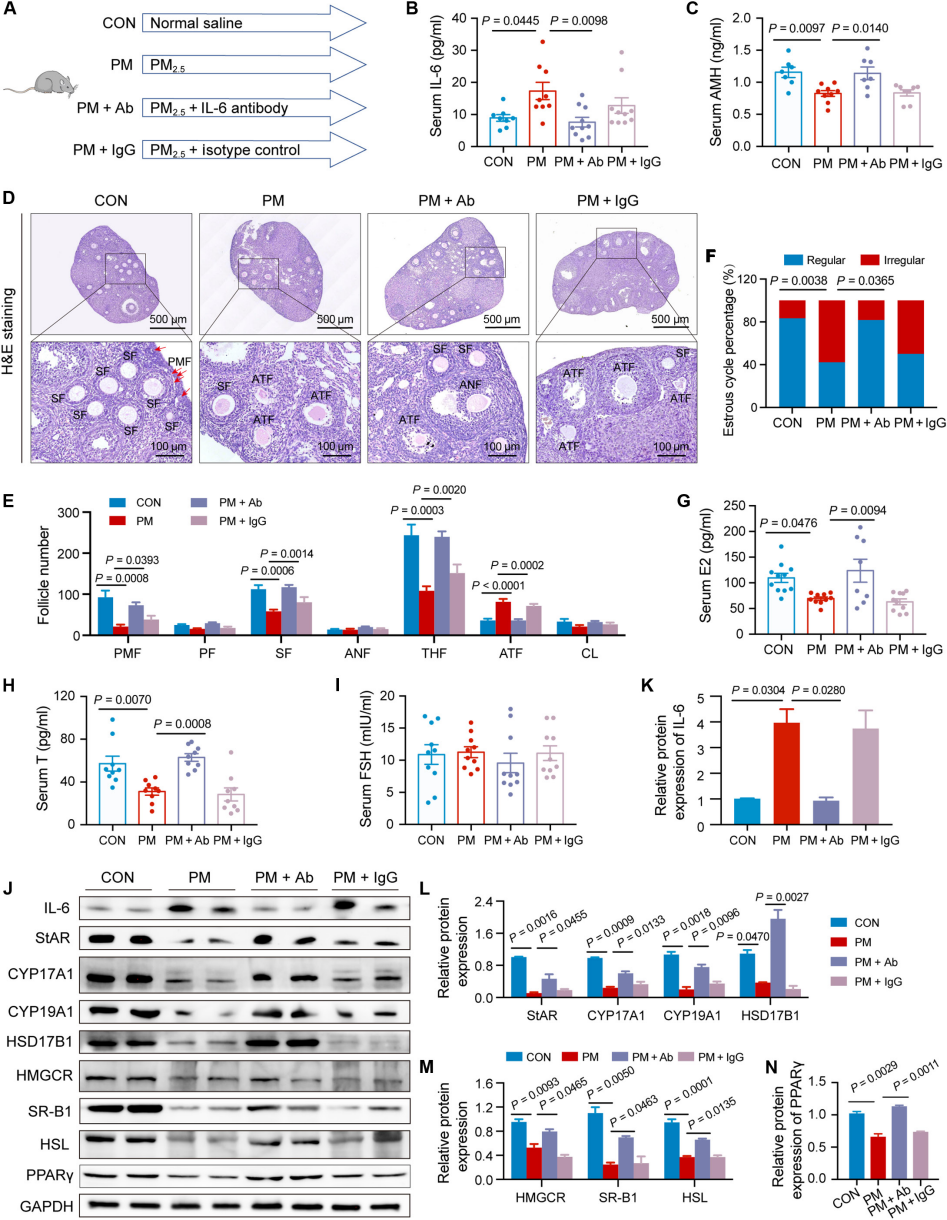
IL-6-neutralizing antibody alleviated PM_2.5_-induced ovarian injury. (A) Scheme of the animal study for assessing whether IL-6-neutralizing antibodies can reverse PM_2.5_-induced ovarian injury. (B) The serum IL-6 level of mice exposed to PM_2.5_ with or without IL-6 antibody administration (*n* = 8 to 10 mice/group, one-way ANOVA). (C) The serum AMH level of mice exposed to PM_2.5_ with or without IL-6 antibody administration (*n* = 7 to 9 mice/group, one-way ANOVA). (D) Ovarian H&E staining images of mice exposed to PM_2.5_ with or without IL-6 antibody administration. (E) Follicle counting results according to ovary serial sections (*n* = 5 mice/group, one-way ANOVA). (F) The proportion of regular or irregular estrous cycles of mice exposed to PM_2.5_ with or without IL-6 antibody administration (*n* = 11 mice/group, chi-square test). (G to I) The serum hormone level of mice exposed to PM_2.5_ with or without IL-6 antibody administration (*n* = 8 to 11 mice/group, one-way ANOVA). (J to N) Protein expression of hormone-synthesis-related genes in ovaries from mice exposed to PM_2.5_ with or without IL-6 antibody administration detected by Western blot (*n* = 3 biological replicates/group, one-way ANOVA).

The above results showed that IL-6 mediates ovarian injury caused by PM_2.5_ exposure. To determine whether IL-6 Abs could ameliorate PM_2.5_-induced ovarian injury, mice were treated with IL-6 Abs (Fig. 6A). The results showed that IL-6 Abs successfully reversed the elevated circulating IL-6 levels caused by PM_2.5_ exposure (Fig. 6B) and alleviated the decline in serum AMH levels and healthy follicle counts (Fig. 6C to E). Moreover, IL-6 Abs ameliorated PM_2.5_-induced estrous cycle disorder, serum E2 and T level decline (Fig. 6F to I), and hormone-synthesis-related gene and PPARγ disruption (Fig. 6J to N). Overall, these results indicate that IL-6 Abs alleviate PM_2.5_-induced ovarian injury, and IL-6 may represent a therapeutic target for the treatment of ovarian damage induced by PM_2.5_.

### Negative correlation of circulating IL-6 levels with ovarian function in women

**Fig. 7.**
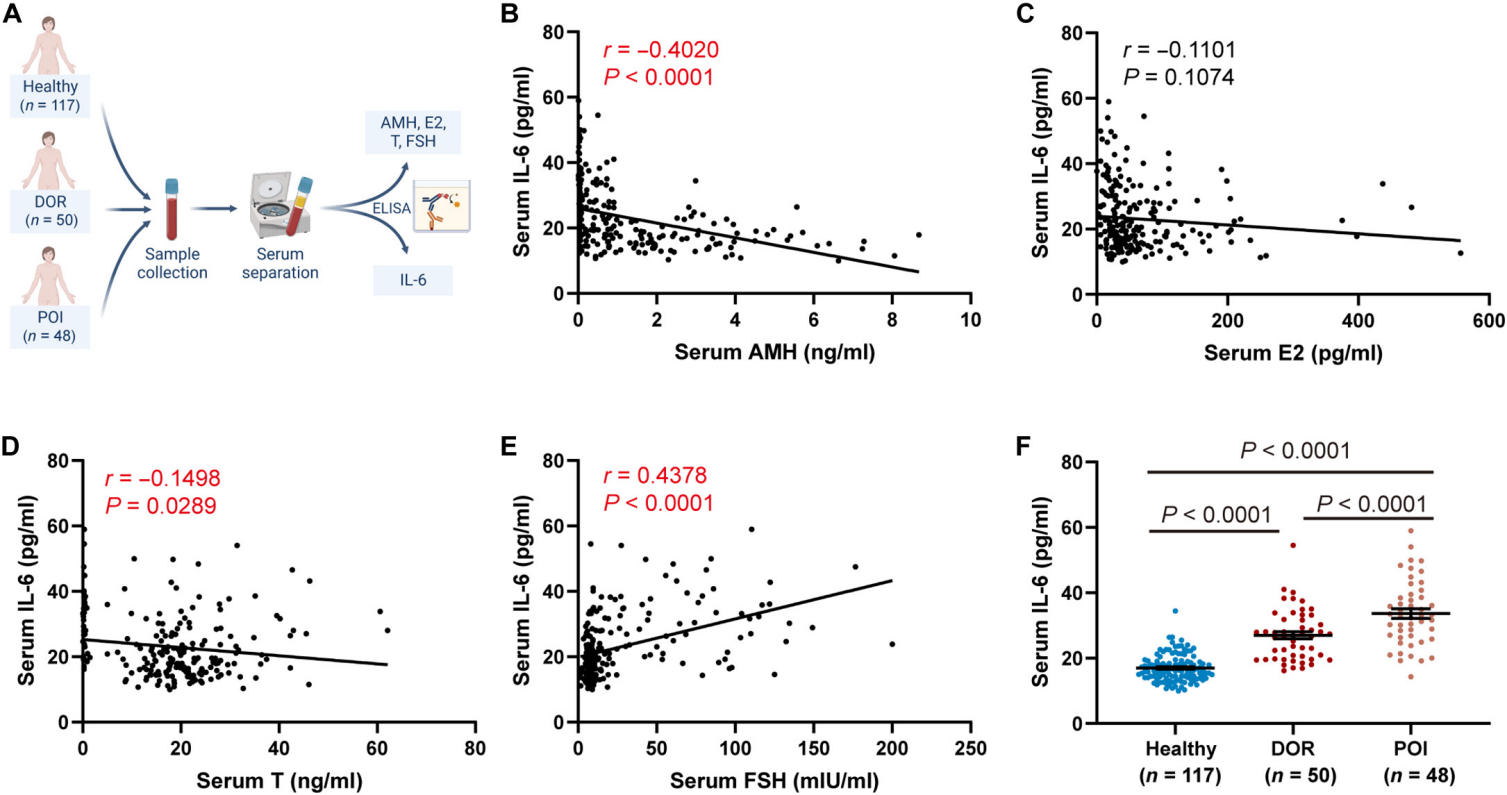
Circulating IL-6 levels correlated with ovarian function in women. (A) Scheme of the population study for assessing the association between circulating IL-6 levels and ovarian function. (B to E) Relationship between serum IL-6 levels and serum levels of AMH, E2, T, and FSH (*n* = 215 participants, Pearson test). (F) Serum IL-6 levels in participants (*n* = 118 participants for healthy women, *n* = 50 participants for DOR women, *n* = 47 patients for POI women, one-way ANOVA). DOR, diminished ovarian reserve; POI, premature ovarian insufficiency.

Premature ovarian insufficiency (POI) and diminished ovarian reserve (DOR) are different phenotypes of premature ovarian aging. The relationship between circulating IL-6 levels and ovarian function was explored in 215 participants consisting of healthy women (*n* = 118), women with DOR (*n* = 50), and women with POI (*n* = 47) (Fig. 7A). The association between serum IL-6 and ovarian steroid hormone levels was analyzed. Serum IL-6 levels were negatively associated with serum levels of AMH (*r* = −0.4020, *P* < 0.0001; Fig. 7B) and T (*r* = −0.1498, *P* = 0.0289; Fig. 7D) and positively associated with serum FSH levels (*r* = 0.4378, *P* < 0.0001; Fig. 7E). No significant association between serum IL-6 levels and concentrations of serum E2 was identified (*r* = −0.1101, *P* = 0.1074; Fig. 7C). Additionally, serum IL-6 levels were significantly higher in patients with DOR and POI than in healthy women (*P* < 0.0001; Fig. 7F). These results demonstrate a negative correlation between circulating IL-6 levels and ovarian function, which suggests that circulating IL-6 may serve as a biomarker for assessing ovarian function.

## Discussion

**Fig. 8.**
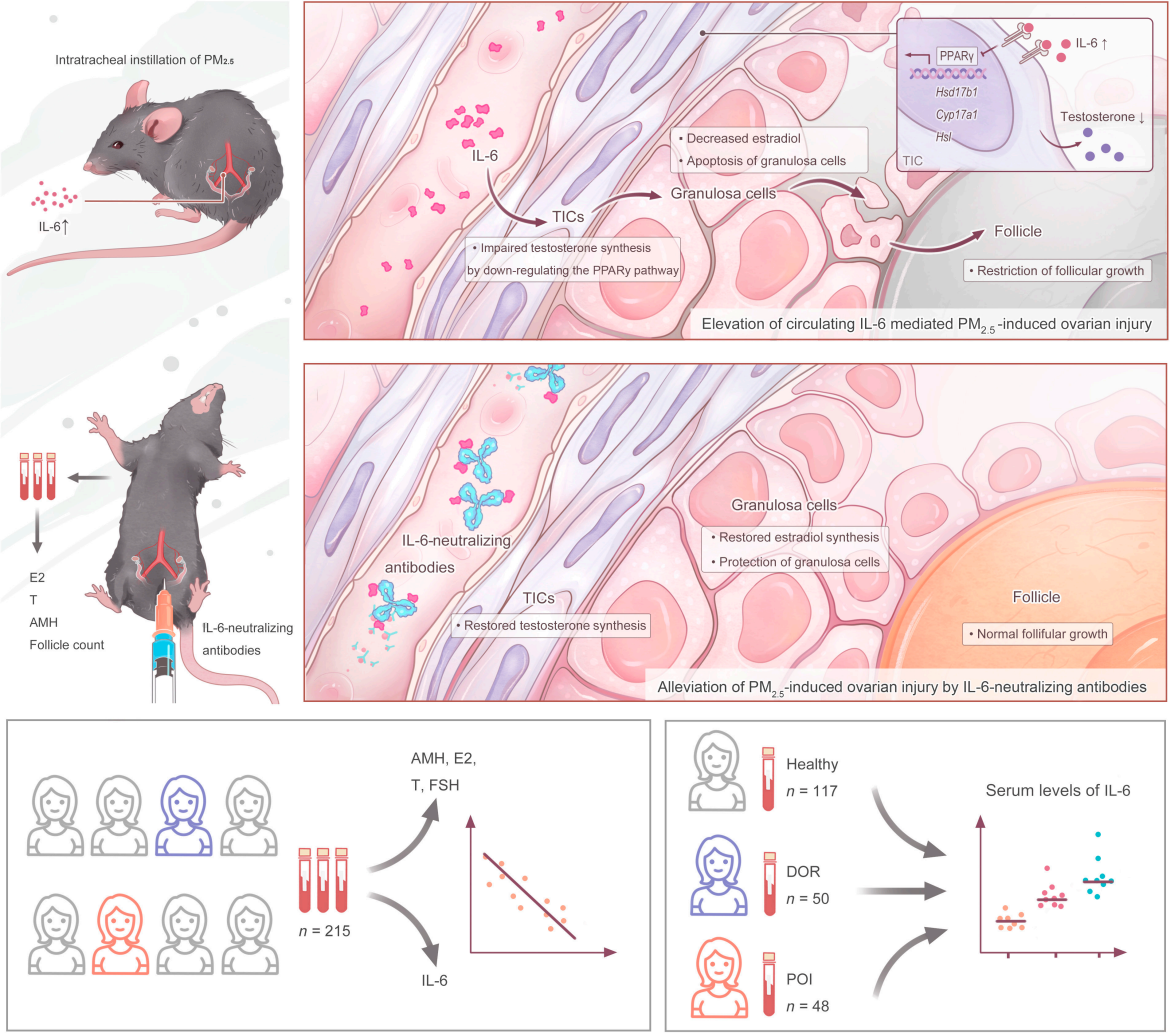
The schematic diagram of the study. PM_2.5_ exposure-induced circulating IL-6 elevation targets TICs and impairs their testosterone synthesis via suppressing the PPARγ pathway, which subsequently restricts estradiol synthesis of granulosa cells and follicular growth. Neutralization of IL-6 effectively ameliorates PM_2.5_-induced ovarian damage. Circulating IL-6 is negatively correlated with ovarian function in woman, and serum IL-6 is significantly increased in DOR and POI patients.

In this study, we demonstrated that PM_2.5_ exposure induces ovarian injury by elevating circulating IL-6, which may not originate from alveolar macrophages alone. Neutralization of IL-6 ameliorates PM_2.5_-induced ovarian damage. We also revealed that circulating IL-6 is negatively correlated with ovarian function and that serum IL-6 is increased in DOR and POI patients. Our findings confirm that IL-6 is a key molecule mediating PM_2.5_-induced ovarian injury, suggesting that it may serve as a predictive marker and therapy target for ovarian impairment (see the schematic diagram of this study in Fig. 8).

Over the past decades, rapid industrialization and urbanization have brought about unavoidable environmental problems. The annual average PM_2.5_ concentration in China in 2021 was 31 μg/m^3^, which meets China’s annual average PM_2.5_ standard (35 μg/m^3^) but below the World Health Organization air quality standard (annual average of 5 μg/m^3^). In the present study, we simulated exposure to PM_2.5_ at 35 μg/m^3^ to identify the cumulative results of sustained PM_2.5_ exposure on ovarian function under relatively good air conditions. Our work demonstrates that PM_2.5_ exposure induces ovarian reserve decline and endocrine dysfunction. These results can serve as a reference for formulating air quality standards.

PM_2.5_ primarily comprises insoluble carbon black particles, adsorbed organic matter, inorganic salts, and heavy metals. A study by Li et al. used fluorescent polystyrene latex microspheres to simulate PM_2.5_ carbon particles and observed their deposition in the lungs and extrapulmonary organs, which showed a nonuniform deposition pattern. At extremely high levels of particle exposure (1,750 μg/m^3^), only single particles were observed in the kidney and liver (organs with abundant blood flow) [36]. However, no direct evidence currently supports the deposition of PM_2.5_ in ovarian tissues. Compounding this issue is the limited blood flow to the ovaries, which renders existing methodologies inadequate for observing the quantity and real-time deposition of PM_2.5_ during typical exposure patterns. Our previous findings have indicated that PM_2.5_ induces IL-6 expression in GCs through the nuclear factor kappa B/IL-6 signaling pathway, which further lead to ovarian injury [20]. However, the direct effects of PM_2.5_ on the cumulus cell–oocyte complex are insufficient to comprehensively account for the ovarian injury. Systemic inflammation has been closely linked to ovarian function [24,25]. Thus, we assume that systemic factors may play a crucial role in ovarian injury caused by PM_2.5_ exposure. Our findings demonstrated that PM_2.5_ exposure induced a systemic inflammatory response, which resulted in elevated levels of circulating IL-6 and keratinocyte-derived chemokine, with IL-6 exhibiting the most pronounced elevation. Furthermore, an analysis of circulating IL-6 levels in women revealed a negative correlation with indicators of ovarian function. Furthermore, rm IL-6 induced ovarian damage, and IL-6 Abs successfully alleviated PM_2.5_-induced ovarian damage. These findings confirmed the critical role of the circulating IL-6 in mediating PM_2.5_-induced ovarian injury.

Additionally, we explored the sources of circulating IL-6. IL-6 can be secreted by various immune cells, such as monocytes, macrophages, lymphocytes, mast cells, and dendritic cells, as well as nonimmune cells, including fibroblasts, endothelial cells, adipocytes, and keratinocytes [37,38]. Unlike other cytokines, IL-6 can exert its effects far away from its source, depending on the level of the circulating IL-6. Previous studies have shown that short-term PM_2.5_ exposure induces IL-6 secretion by alveolar macrophages, which leads to elevated circulating IL-6 levels. The clearance of alveolar macrophages reverses the elevated circulating IL-6 levels and thrombosis caused by PM_2.5_ exposure [27–29]. However, in our study, the clearance of alveolar macrophages neither alleviated the PM_2.5_-induced increase in circulating IL-6 levels nor improved ovarian function, suggesting that alveolar macrophages are not the primary source of circulating IL-6 in this long-term exposure model. The inconsistency may be due to differences in PM_2.5_ composition, exposure dose, and exposure time. First, the PM_2.5_ particles used in our study were collected in Wuhan, China, while Budinger’s group’s studies were conducted in Chicago [27,28]. The composition of PM_2.5_ varies from region to region [39,40]. Second, the dose explored in this study was equivalent to a human exposure concentration of 35 μg/m^3^, while the exposure concentrations were 109.1 ± 6.18 and 118.3 ± 5.21 μg/m^3^ in Budinger’s group’s studies [27,29]. Finally, mice were exposed to PM_2.5_ for 4 weeks in this study, while the exposure period was 24 h in the studies mentioned above [27–29]. The chronic and acute exposure patterns likely result in different responses of immune cells to PM_2.5_.

We further explored the mechanisms by which IL-6 mediates ovarian injury caused by PM_2.5_. Although IL-6 has been extensively studied in autoimmune diseases, infections, and tumors [41], little research has been conducted on its role in ovarian function. Suriyakalaa et al. [42] showed that IL-6 affects androgen synthesis in bovine TICs by inhibiting the expression of LHCGR, StAR, and CYP17A1. In this study, we found that IL-6 affected the expression of the hormone-synthesis-related genes, which are CYP17A1, HSD17B1, and HSL. RNA sequencing suggested that the down-regulated genes were enriched in the PPAR pathway. PPARγ, a nuclear transcription factor, heterodimerizes with the 9-*cis*-retinoic acid receptor (RXR) upon ligand activation and promotes target gene expression by binding to DNA reaction elements. PPARγ has been proved to regulate the expression of CYP19A1, HSD3B, and HSD17B [43]. The PPARγ agonist, GW1929, can increase serum estrogen levels, decrease FSH and luteinizing hormone levels, and reduce ovarian apoptosis by down-regulating BAX expression and up-regulating BCL2 expression in perimenopausal rats [44]. In our study, IL-6 affected the expression of key genes (CYP17A1, HSD17B1, and HSL) involved in hormone synthesis through the down-regulation of the PPARγ pathway, resulting in decreased hormone synthesis capacity.

Notably, circulating IL-6 levels negatively correlated with ovarian function in women. Previous studies have demonstrated a positive correlation between follicular fluid IL-6 levels and female age, whereas low levels of follicular fluid IL-6 are associated with increased success in in vitro fertilization clinical pregnancies [45]. Circulating IL-6 levels were significantly higher in postmenopausal women than in nonmenopausal women [46]. In addition, circulating IL-6 levels were significantly higher in patients with premature ovarian failure than in normal controls [25]. Our results revealed that circulating IL-6 levels were negatively associated with serum AMH and T levels and positively associated with serum FSH levels in women. Serum IL-6 levels were significantly higher in patients with DOR or POI than in healthy participants. Moreover, IL-6 Abs ameliorated ovarian injury induced by exposure to PM_2.5_ in mice. Based on the literature and our findings, circulating IL-6 is a predictive biomarker and therapeutic target for ovarian injury.

In summary, our research provides experimental evidence that exposure to PM_2.5_ induces ovarian injury by elevating circulating IL-6, which targets TICs and impairs T synthesis via the PPARγ pathway, which further affects GC function and follicle development. Alveolar macrophages are not the major source of elevated IL-6. Our results indicate that IL-6 may be a biomarker for premature ovarian aging and a potential target for the treatment of PM_2.5_-related ovarian injury. Our findings offer new insights into the understanding of PM_2.5_-induced ovarian damage, presenting a promising avenue for evaluating ovarian function and developing clinical strategies for alleviating ovarian damage.

Our research findings suggest that serum IL-6 severely impacts ovarian function, with the source of IL-6 likely being diverse. While previous studies have suggested that PM_2.5_ can enhance IL-6 secretion by alveolar macrophages, our study corroborated this observation. Nevertheless, the restoration of ovarian function was not achieved by eliminating alveolar macrophages, suggesting that IL-6 may be produced by various systems. Further investigation is necessary to evaluate the effectiveness of IL-6 antibodies in preventing ovarian damage in nonhuman primates, which has profound implications for future translational research.

## Materials and Methods

### Human samples

Overall, 118 healthy women with normal ovarian function, 50 patients with DOR, and 47 patients with POI were recruited from Tongji Hospital, Tongji Medical College, Huazhong University of Science and Technology (Wuhan, China) between 2019 and 2022. Healthy women had regular menses and normal serum ovarian steroid hormone levels. The patients with DOR met the following diagnostic criteria: a decrease in the number and/or quality of oocytes in the ovary and/or a decrease in the level of AMH (<1.1 ng/ml) and/or a decrease in AFC (<6) and/or an increase in the level of basal FSH (>10 U/l). The patients with POI met the diagnostic criteria for POI: (a) age < 40 years, with amenorrhea or scanty menstruation ≥4 months, with or without hypoestrogenic symptoms (hot flashes, palpitation, and insomnia), and (b) 2 serum FSH measurements >25 U/l, taken at an interval of >4 weeks. Both criteria must be satisfied simultaneously. The exclusion criteria were malignant tumors, severe infection, severe liver or kidney dysfunction, prior removal of the uterus or ovary, and use of medications known to affect the immune status or serum levels of ovarian steroid hormones, including oral contraceptives, lipid-lowering agents, antihypertensive drugs, functional foods, and vitamin or mineral supplementation. This study was approved by the ethics committee of Tongji Hospital (No. TJ-IRB20210319). Written informed consent was obtained from all participants at enrollment.

### Animals

Female C57BL/6 mice (8 weeks old) were purchased from GemPharmatech (China) and fed ad libitum for 1 week for environmental acclimation. All mice were maintained in specific-pathogen-free conditions with filtered air at a constant temperature of 22 ± 2 °C and a relative humidity of 55% ± 10% with a 12:12 h light:dark cycle throughout the study. All animal studies were conducted in accordance with the principles of laboratory animal care.

### Ethics statement

All animal procedures and protocols were approved by the ethics committee of Tongji Hospital, Tongji Medical College, Huazhong University of Science and Technology of the People’s Republic of China (No. TJH-202201023).

### PM_2.5_ collection

PM_2.5_ was gathered by Teflon membrane filters (Whatman, USA) with a PM_2.5_ air sampler (TH-150D, Tianhong, Wuhan, China) in Tongji Hospital, Huazhong University of Science and Technology, Wuhan, China (114.12119°E, 30.542089°N) and was extracted from the Teflon membrane filters using the methodology of previous studies [47,48]. Briefly, to scatter the PM_2.5_, the filters were ultrasonicated for 30 min with a sonicator (KQ-700 V, Shumei, China) in deionized water (18 MΩ/cm). Subsequently, the suspensions were freeze-dried to form PM_2.5_ powder. The PM_2.5_ sample was stored at −80 °C for later use.

### Exposure to PM_2.5_

The objective of this study was to evaluate the effects of PM_2.5_ exposure on ovarian function. Two groups of C57BL/6 mice (22 mice per group) were used for the animal experiments. The mice in the CON group were treated with vehicle (saline), while those in the PM group were treated with PM_2.5_ suspension (8 mg/kg bw) via intratracheal instillation every 2 d. The PM_2.5_ exposure dose was calculated based on the physiological parameters of the mice. The tidal volume of the mice was approximately 0.15 ml, and the respiratory rate was approximately 150 breaths/min. Therefore, the respiratory volume of the mice was approximately 32.4 l/d (0.15 ml × 150 breaths/min × 60 min × 24 h = 32.4 l). Based on the annual average standard of PM_2.5_ in China (35 μg/m^3^), the exposure dose of PM_2.5_ was estimated to be 0.7938 μg/d (32.4 l × 35 μg/m^3^ × 70% = 0.7938 μg), because approximately 70% of inhaled PM_2.5_ is deposited in the deep airways [49,50]. Taking into account a 100-fold uncertainty factor [51,52], an intervention concentration of 8 mg/kg bw PM_2.5_ was set for this experiment (0.7938 μg × 100 × 2 d ÷ 20 g = 7.938 mg/kg bw ≈ 8 mg/kg bw), which is in line with the doses used in previous studies [20,53]. After 4 weeks of exposure, blood samples were collected from the angular vein, and the mice were sacrificed. Six ovaries were fixed in 4% paraformaldehyde at 4 °C. The remaining ovaries were stored in liquid nitrogen for further messenger RNA or protein detection.

### rm IL-6 treatment

This study investigated the correlation between circulating IL-6 levels and ovarian injury. Two groups of C57BL/6 mice were used in the animal experiments (16 mice per group). The CON group received vehicle treatment (normal saline), whereas the experimental group (rm IL-6) received rm IL-6 (400 ng/mouse, HY-P7063, MedChemExpress, USA) via intraperitoneal injection every 2 d. The treatment duration for both groups was 4 weeks.

### Clodronate liposome treatment

This study determined whether circulating IL-6 originates from alveolar macrophages. This batch of animal experiments included 4 groups, each consisting of 16 C57BL/6 mice. The groups consisted of the following treatments: mice treated with vehicle (normal saline) as the CON group, mice treated with PM_2.5_ suspension (8 mg/kg bw) by intratracheal instillation every 2 d (PM), mice treated with PM_2.5_ suspension (8 mg/kg bw) every 2 d and clodronate liposomes (50 μl/mouse, C-005, LIPOSOMA, Netherlands) every 4 d by intratracheal instillation (PM + CLS), and mice treated with clodronate liposomes (50 μl/mouse) by intratracheal instillation every 4 d (CLS). The mice were treated for 4 weeks.

### IL-6 Ab treatment

This study aimed to explore whether IL-6 Abs could alleviate PM_2.5_-induced ovarian injury. The commercial antimouse IL-6 Ab has been extensively validated in preclinical mouse studies [54–57]. This batch of animal experiments included 4 groups, 16 C57BL/6 mice per group. The mice were treated with vehicle (normal saline) (CON group), PM_2.5_ suspension (8 mg/kg bw) by intratracheal instillation every 2 d (PM), PM_2.5_ suspension (8 mg/kg bw) by intratracheal instillation every 2 d and IL-6 antibodies (100 μg/mouse, A2118, Selleck, USA) by intraperitoneal injection twice a week (PM + IL-6 Ab), and PM_2.5_ suspension (8 mg/kg bw) by intratracheal instillation every 2 d and immunoglobulin G (IgG) isotype (100 μg/mouse, A2119, Selleck, USA) by intraperitoneal injection twice a week (PM + IgG). The treatment duration for all groups was 4 weeks.

### Bronchoalveolar lavage fluid collection

Mice were secured in the supine position on the operating table after anesthesia. The skin on the neck and chest was disinfected using 75% alcohol. The trachea was exposed by cutting off the sternum after a cut on the neck skin. An approximately 0.2-cm-long incision was then made obliquely in the proximal part of the trachea. The medical indwelling needle was inserted into an approximately 1-cm-deep proximal part, and the incision was ligated and fixed using silk threads. Subsequently, the lungs were lavaged with 0.5 ml of cold phosphate-buffered saline (PBS) for 3 cycles each. Subsequently, 1 ml of bronchoalveolar lavage fluid was fully recycled. To measure the IL-6 levels, the supernatant was centrifuged and aspirated.

### Isolation and culture of GCs and TICs

Mouse GCs and TICs were collected by gently puncturing the preantral follicles from 21- to 23-d-old mice using a 25-G syringe needle, as previously described [58]. TICs were pretreated with graded concentrations of rm IL-6 (0 to 50 ng/ml, HY-P7063, MedChemExpress, USA) for 24 h. Afterward, they were washed thrice with PBS and incubated in a complete medium without IL-6 for another 24 h. Culture supernatants from the TICs were collected and used for GC culture.

### Isolation and culture of prenatal follicles

Follicle culture methods have been described previously [59]. The ovaries of 2-week-old mice were removed by blunt dissection in L15 medium (PYG-0038, Boster, Wuhan, China). Following mechanical dissection using 2 syringe needles, only the follicles with an intact basal membrane, central and spherical oocytes, and intact GCs were selected for follicle culture in 100 μl of culture medium at 37 °C and 5% CO_2_. For the follicle culture, the conditioned medium from TICs was used. Refreshments were performed every other day by removing and replacing 50 μl of the medium. Before the medium refreshment, photographs of every follicle were taken using an inverted microscope (Olympus, Tokyo, Japan).

### Estrous cycle detection

Vaginal smears were collected every morning at 0900 for 14 consecutive days using 10 μl of normal saline placed on glass slides. The slides were stained with H&E before microscopic examination to determine the cell cycle phase.

### Follicle counting

The ovaries were fixed in 4% paraformaldehyde overnight, dehydrated in an ethanol gradient, and embedded in paraffin. Serial sections of 4-μm thickness were then obtained. The 4 sections were mounted on glass slides. H&E staining was performed on every fourth slide. Two individuals, blinded to the origin of the sections, analyzed the stained slides under a microscope. Only the follicles containing oocytes were included in the follicle count. This procedure is based on previous descriptions [20,60].

### Enzyme-linked immune sorbent assay

Blood samples were collected during estrus. The levels of AMH, E2, T, and FSH were measured by enzyme-linked immune sorbent assay according to the manufacturer’s instructions (CSB-E13156m, CSB-E05109, CSB-E05101, and CSB-E06871, Cusabio, China). IL-6 levels were measured using enzyme-linked immune sorbent assay according to the manufacturer’s instructions (E-EL-M0044, Elabscience, China).

### Luminex liquid suspension chip detection

Cytokines in the circulation and ovarian tissues were detected using the Luminex liquid suspension chip detection technology offered by Wayen Biotechnologies (Shanghai, China). The Bio-Plex Pro Mouse Cytokine GrpI Panel 23-plex kit was used according to the manufacturer’s instructions. Briefly, murine serum or ovarian tissue homogenates were incubated in 96-well plates containing the microbeads for 1 h. The detection antibody was then added and incubated for 30 min. Streptavidin–phycoerythrin was then added to each well and incubated for 10 min. Finally, the Bio-Plex MAGPIX System (Bio-Rad) was used to read the values.

### Western blot

Ovaries and cells were harvested and lysed in a radioimmunoprecipitation assay buffer. polyvinylidene fluoride membranes were incubated with different primary antibodies such as β-actin (1:5,000, AC004, ABclonal, China), glyceraldehyde-3-phosphate dehydrogenase (1:5,000, AC002, ABclonal, China), HSD3B1 (1:1,000, A19266, ABclonal, China), CYP11A1 (1:1,000, A23954, ABclonal, China), CYP17A1 (1:1,000, A5067, ABclonal, China), CYP19A1 (1:1,000, 16554-1-AP, Proteintech, China), HSD17B1 (1:1,000, A10839, ABclonal, China), NR1H3 (1:1,000, A3974, ABclonal, China), HMGCR (1:1,000, A19063, ABclonal, China), HMGCS1 (1:1,000, A3916, ABclonal, China), SR-B1 (1:1,000, A0827, ABclonal, China), HSL (1:1,000, A24689, ABclonal, China), and IL-6 (1:1,000, 12912, Cell Signaling Technology, USA) overnight at 4 °C. Afterward, the membranes were incubated with the secondary antibody, horseradish peroxidase-conjugated goat antirabbit IgG (H + L) (1:3,000, GB23303, Servicebio, China) for 30 min at 37 °C. The relative amounts of proteins were quantified using Image Lab software (Bio-Rad, USA). β-Actin or glyceraldehyde-3-phosphate dehydrogenase was used as an internal control.

### Immunohistochemistry

The expression of IL-6Rα in ovaries and F4/80, CD3, and CD19 in lungs was detected by IHC staining. Sections were incubated overnight at 4 °C with primary antibodies IL-6Rα (1:100, ab300581, Abcam, USA), F4/80 (1:200, 70076, Cell Signaling Technology, USA), CD3 (1:200, 78588, Cell Signaling Technology, USA), and CD19 (1:200, 90176, Cell Signaling Technology, USA). The following day, sections were rinsed with PBS and incubated with the secondary antibody for 1 h at 37 °C. To visualize IL-6Rα-, F4/80-, CD3-, and CD19-positive cells, diaminobenzidine (G1212-200T, Servicebio, China) was used.

### Transmission electron microscopy

Ovarian samples were prepared for TEM analysis, as previously described [61]. Samples were dehydrated using a series of ascending ethanol concentrations, fixed in osmium tetroxide, and embedded in epoxy resin. Images were captured using an 80-kV TEM instrument (CM 10, Philips, Netherlands).

### Quantitative real-time polymerase chain reactions

Total RNA was extracted using the TRIzol reagent (15596018CN, Invitrogen, USA). Reverse transcription kits (R423-01, Vazyme, Nanjing, China) were used to convert 1 μg of RNA into complementary DNA. Quantitative PCR was conducted using ChamQ Universal SYBR qPCR Master Mix (Q711-02, Vazyme, China) in a CFX96 real-time PCR system (Bio-Rad, USA). The messenger RNA expression level of target genes was calculated using the 2^−ΔΔCt^. The primer sequences are listed in Table S1.

### Cell apoptosis detection by flow cytometry

Apoptosis in GCs was detected by flow cytometry using FITC-Annexin V Apoptosis Detection Kit I (556547, BD Pharmingen, USA), following the manufacturer’s instructions. GCs were harvested using trypsin and washed twice with PBS. Subsequently, the cells were incubated with annexin V–propidium iodide for 15 min. Flow cytometry was performed using a flow cytometer to determine the population of annexin V-positive cells (BD Pharmingen). The percentage of apoptotic cells in the upper and lower right quadrants was calculated.

### Uniform manifold approximation and projection analysis

Murine single-cell RNA sequencing was conducted by our research team. Raw sequencing and processed data are available in the National Center for Biotechnology Information Gene Expression Omnibus under the accession number “GSE241318”. A dimensional reduction plot displaying the distribution of IL-6Rα expression was made using the “FeaturePlot” function of the Seurat package.

### Statistical analysis

The experiments described above were repeated at least thrice, except for the animal experiments. Values are expressed as mean ± standard error of the mean unless noted otherwise. The Q–Q plot and the Shapiro–Wilk test were combined to test the normality of the data using SPSS 24. Statistical significance was assessed using a 2-tailed Student *t* test, a chi-square test, and one-way or 2-way analysis of variance using the GraphPad Prism 7 software. Pearson’s correlation coefficients were used to examine the relationship between serum IL-6 and ovarian steroid hormone levels in women. *P* < 0.05 was considered statistically significant.

## Data Availability

All data are available in the main text or the supplementary materials.
