# Supplementary material for: Circulating Interleukin-6 Mediates PM2.5-Induced Ovarian Injury by Suppressing the PPARγ Pathway
Source: Research (Wash D C). 2024 Dec 5;7:0538. doi: 10.34133/research.0538 (PMC11617621; doi:10.34133/research.0538)
Supplement: Supplementary 1 — Figs. S1 to S3 Table S1 [file research.0538.f1.zip › Table S1.docx]

**Table S1**. The sequences of primers used for qRT-PCR assay.

| Gene | Forward primer (5’-3’) | Reverse primer (3’-5’) |
| --- | --- | --- |
| *Star* | AACGGGGACGAAGTGCTAAG | CCGTGTCTTTTCCAATCCTCTG |
| *Cyp11a1* | AGGTCCTTCAATGAGATCCCTT | TCCCTGTAAATGGGGCCATAC |
| *Cyp17a1* | AGTGCTCGTGAAGAAGGGGA | TTTCCTTGGTCCGACAAGAGG |
| *Hsd3b1* | AGCTCTGGACAAAGTATTCCGA | GCCTCCAATAGGTTCTGGGT |
| *Hsd17b1* | ACTTGGCTGTTCGCCTAGC | GAGGGCATCCTTGAGTCCTG |
| *Hsd17b7* | TCTCTGCCATGTGGATAACCC | GGTCGGTAGCGTATTTGGAAG |
| *Hmgcr* | AGCTTGCCCGAATTGTATGTG | TCTGTTGTGAACCATGTGACTTC |
| *Hmgcs1* | AACTGGTGCAGAAATCTCTAGC | GGTTGAATAGCTCAGAACTAGCC |
| *Sr-b1* | TTTGGAGTGGTAGTAAAAAGGGC | TGACATCAGGGACTCAGAGTAG |
| *Hsl* | GATTTACGCACGATGACACAGT | ACCTGCAAAGACATTAGACAGC |
| *Pparα* | AGAGCCCCATCTGTCCTCTC | ACTGGTAGTCTGCAAAACCAAA |
| *Pparβ* | TCCATCGTCAACAAAGACGGG | ACTTGGGCTCAATGATGTCAC |
| *Pparγ* | TCGCTGATGCACTGCCTATG | GAGAGGTCCACAGAGCTGATT |
| *Gapdh* | TGAACGGGAAGCTCACTGG | TCCACCACCCTGTTGCTGTA |
