# Supplementary figures and images for: Circulating Interleukin-6 Mediates PM2.5-Induced Ovarian Injury by Suppressing the PPARγ Pathway
Source: Research (Wash D C). 2024 Dec 5;7:0538. doi: 10.34133/research.0538 (PMC11617621; doi:10.34133/research.0538)

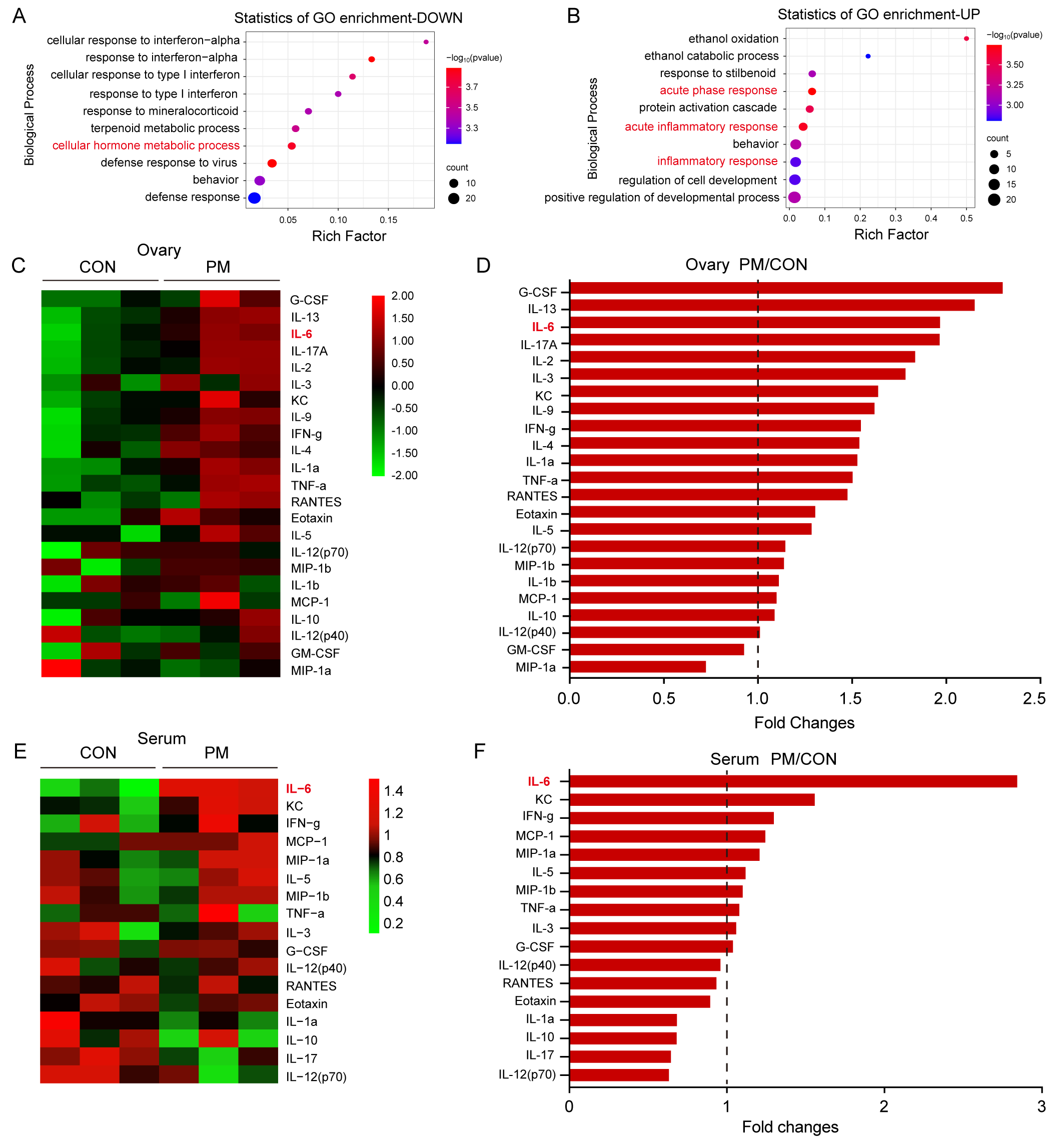

Supplement: Supplementary 1 — Figs. S1 to S3 Table S1 [file research.0538.f1.zip › Fig S1.tif]

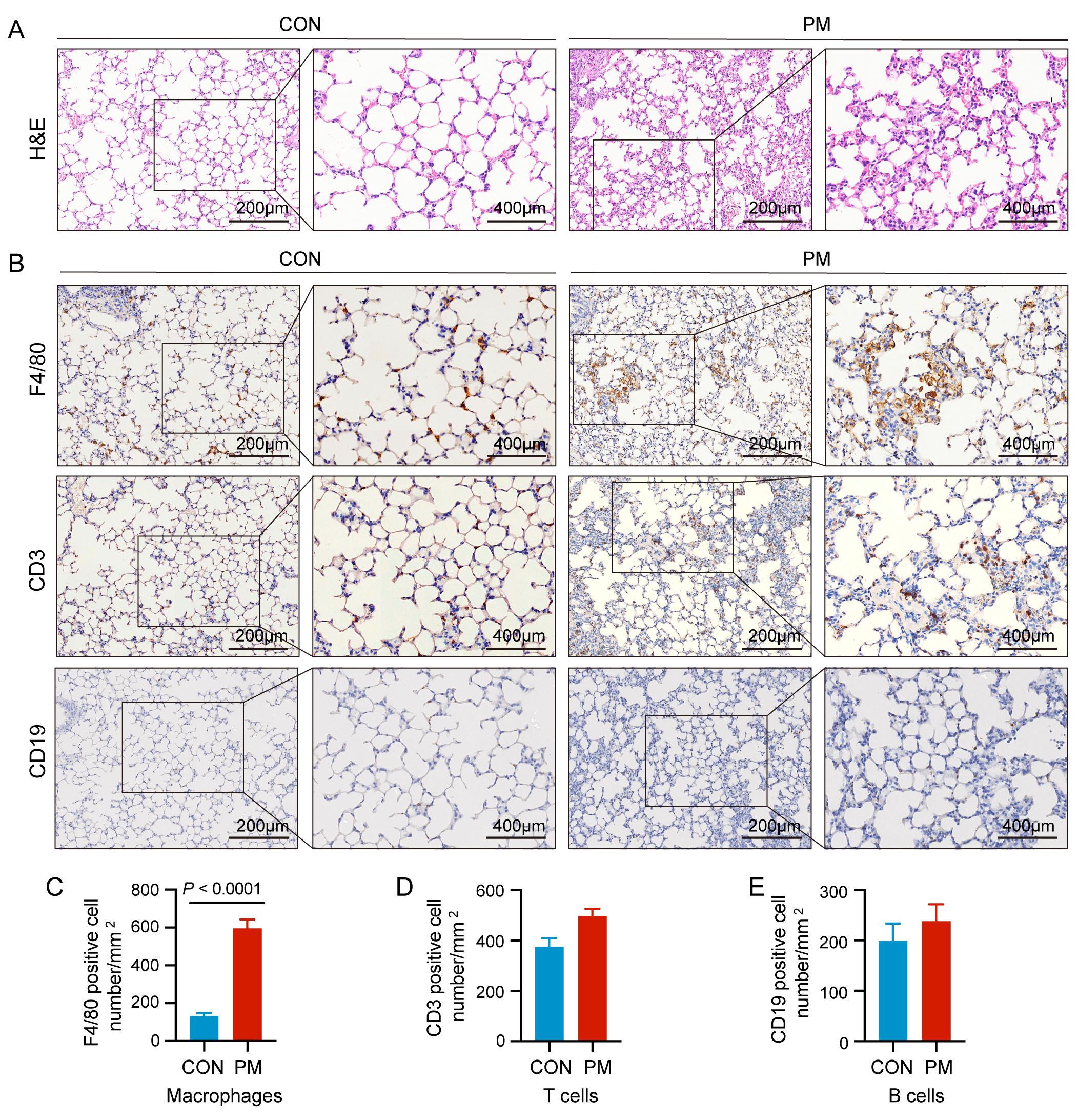

Supplement: Supplementary 1 — Figs. S1 to S3 Table S1 [file research.0538.f1.zip › Fig S2.tif]

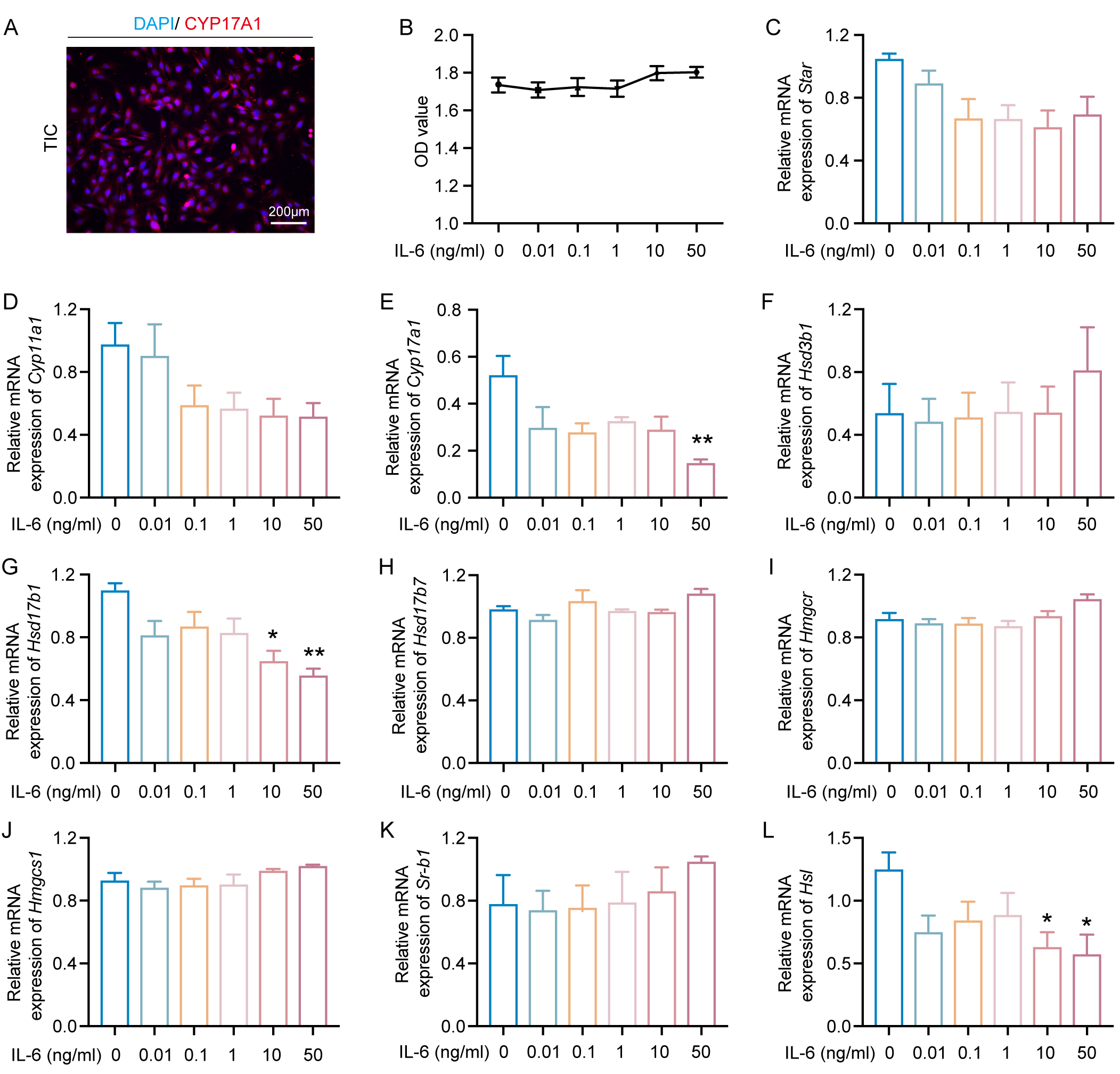

Supplement: Supplementary 1 — Figs. S1 to S3 Table S1 [file research.0538.f1.zip › Fig S3.tif]
